# Catastrophic Cardiac Complications of Takayasu’s Arteritis

**DOI:** 10.7759/cureus.9142

**Published:** 2020-07-11

**Authors:** Sara A Godil, Bilal Saqi, Kareem Godil, Syed Rafay Ali Sabzwari, Yasotha Rajeswaran

**Affiliations:** 1 Internal Medicine, Lehigh Valley Health Network, Allentown, USA; 2 Cardiology, Lehigh Valley Health Network, Allentown, USA

**Keywords:** coronary artery bypass grafting(cabg), coronary artery angiography, aortic valve insufficiency, mitral valve surgery, takayasu disease, systolic heart failure

## Abstract

Takayasu's arteritis (TA) causes inflammation and necrosis of vessel walls, leading to aneurysm formation, extensive coronary damage and valvular abnormalities. We review a case of recurrent coronary, aortic and mitral valve involvement in a patient with TA and discuss the various treatment options available for such patients.

## Introduction

Takayasu's arteritis (TA), also known as pulseless disease, is a chronic inflammatory arteritis involving large vessels of the body. It typically presents in the second and third decades of life with a predilection for females [1,2]. The exact etiology is unclear, but studies have shown an immunologic response triggering a subset of T and B lymphocytes and macrophages that leads to acute inflammation and necrosis of vessel walls, leading to stenosis and aneurysms [3]. We present a case of a 29-year-old female with a history of TA causing extensive and recurrent coronary disease, valvular damage and aortic root involvement and review the pathogenesis along with the medical and surgical options currently available.

## Case presentation

A 29-year-old female presented to the emergency department with a four-week history of worsening dyspnea on exertion, lower extremity edema and a nine-pound weight gain. On arrival, her vital signs were stable. Physical exam revealed a late peaking systolic ejection murmur, jugular venous distension and 2+ lower extremity edema bilaterally. Blood work was significant for troponin I of 0.52 ng/ml and N-terminal pro B-type natriuretic peptide (NT-proBNP) of 21K pg/ml. Electrocardiogram showed sinus tachycardia. An echocardiogram nine months ago had reported moderate left ventricular hypertrophy with an ejection fraction (EF) of 60%, a well-seated bioprosthetic aortic valve with prosthetic valve stenosis (mean gradient [MG] 33 mmHg, valve area 0.8 cm^2^) and a well-seated mitral valve (MV) (MG 5 mmHg).

The patient has a history of hypertension and TA diagnosed at the age of 19 years. The patient was started on prednisone and methotrexate, which she self-discontinued after a few months. Five months later, the patient developed symptoms of heart failure and underwent an echocardiogram and a left heart catheterization (LHC). The patient was found to have severe stenosis of proximal left main coronary artery with an EF of 20%, severe aortic and mitral insufficiency, and a dilated ascending aorta (4 cm). She underwent coronary artery bypass graft (CABG) (left internal thoracic artery to the diagonal branch), replacement of ascending aorta (26 Medox graft; Meadox Medicals, Inc, Oakland, NJ), aortic valve replacement (AVR) (23 Carpentier-Edwards pericardial valve; Edwards Lifescience, Irvine, CA) and MV annuloplasty (26 Physio annuloplasty ring; Edwards Lifescience, Irvine, CA). She decided against mechanical valve as she did not want to be on lifelong anticoagulation. She was started on tocilizumab, which she discontinued a year prior to this presentation.

**Figure 1:**
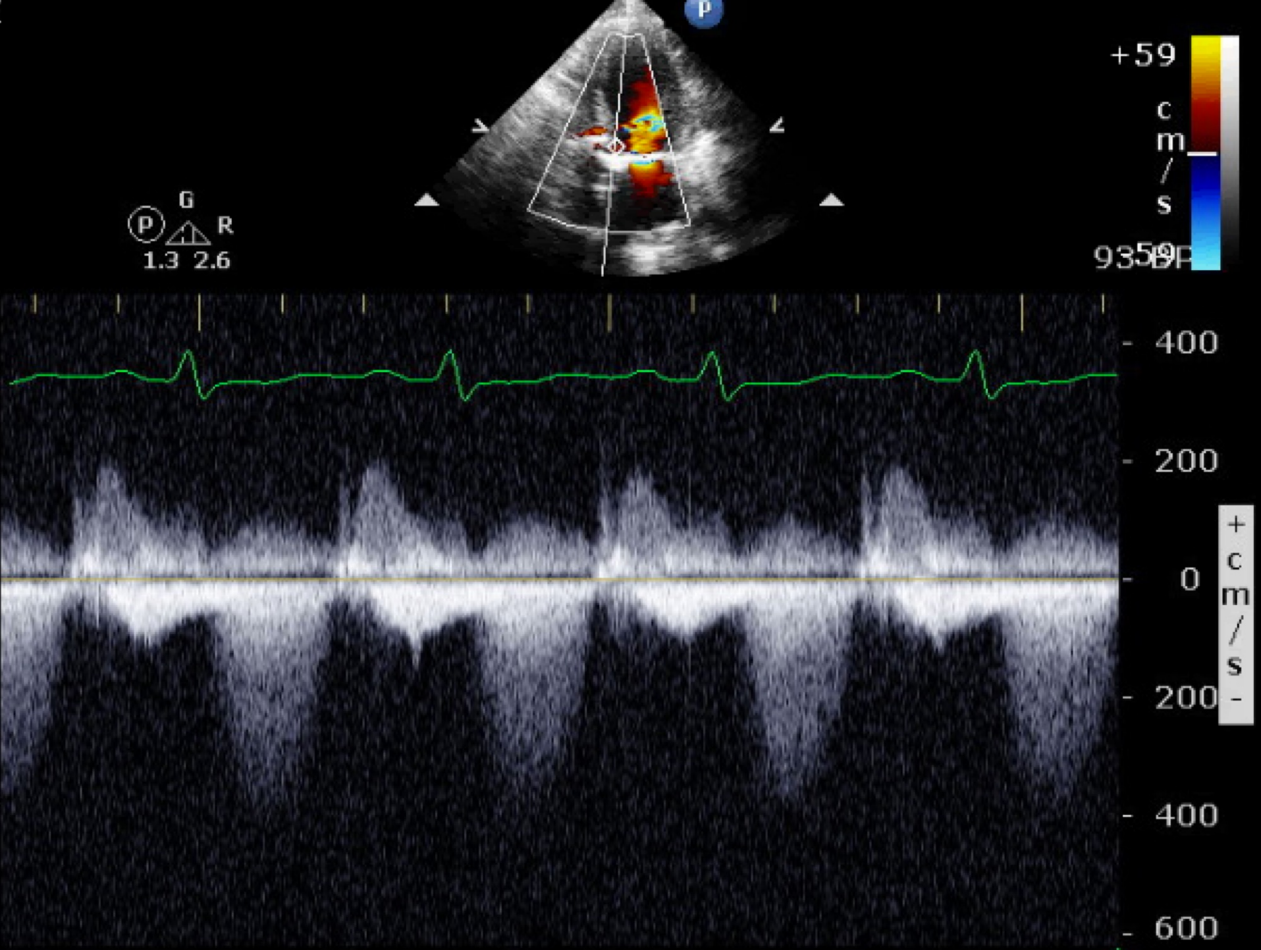
Transthoracic echocardiogram showing continuous wave Doppler signal across the prosthetic aortic valve with a peak velocity of 4.1 m/s.

On this admission, a repeat echocardiogram was done, which showed global hypokinesis with severely reduced left ventricular systolic function and EF of 30%, severe prosthetic aortic valve stenosis (MG 34.3 mmHg, valve area 0.7 cm^2^, acceleration time 120 ms, dimensionless index 0.2, indexed effective orifice area 0.4 cm^2^/m^2^) (Figure 1) and possible MV stenosis (MG 7.4 mmHg, valve area 1.4 cm^2^, peak velocity 2.51 m/s).

**Figure 2:**
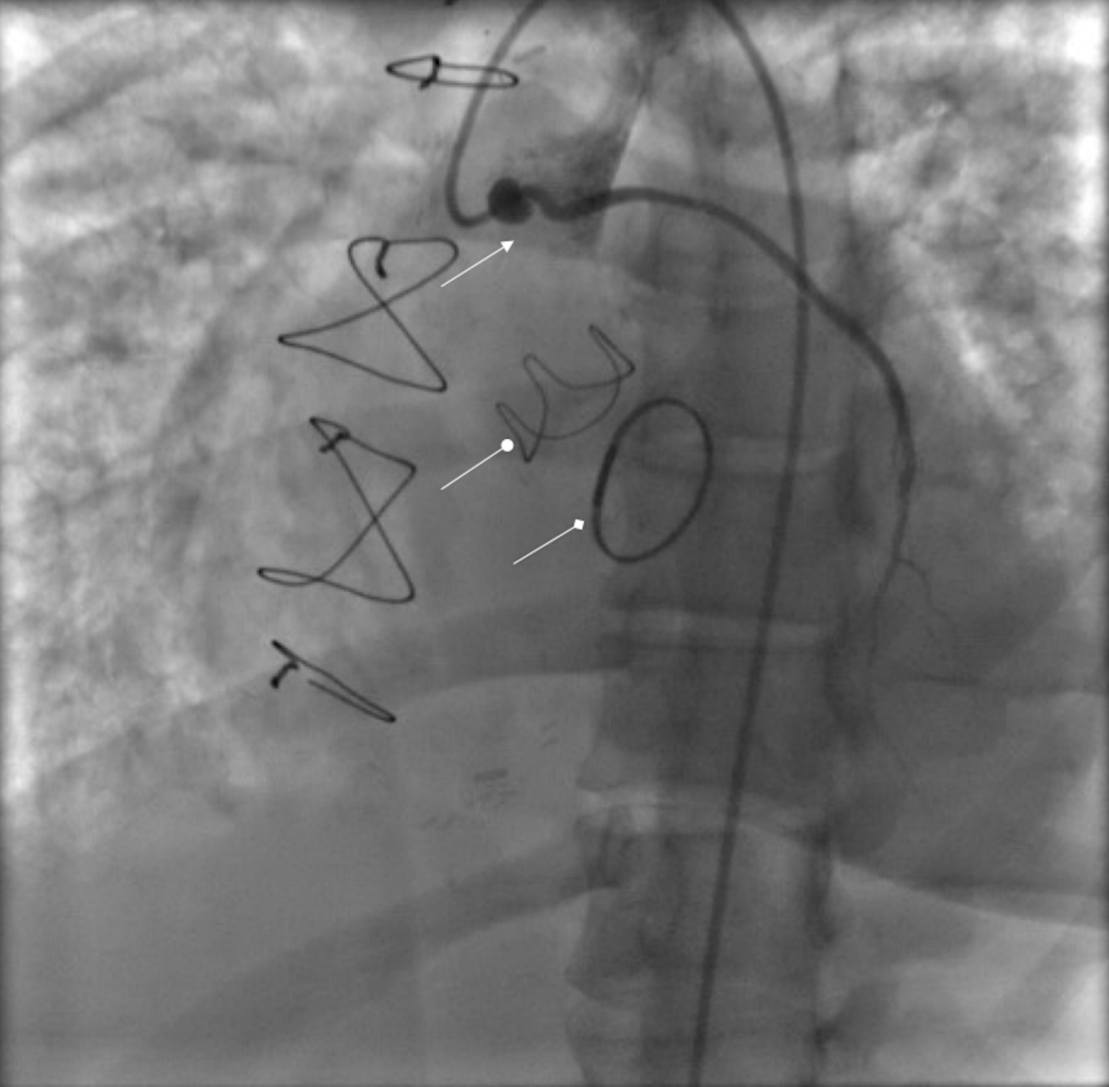
Left heart catheterization showing a patent graft to the second diagonal artery (arrow). The graft is aneurysmal at proximal anastomosis. Prosthetic aortic valve (oval arrow) and mitral repair ring (diamond arrow) also visualized.

**Figure 3:**
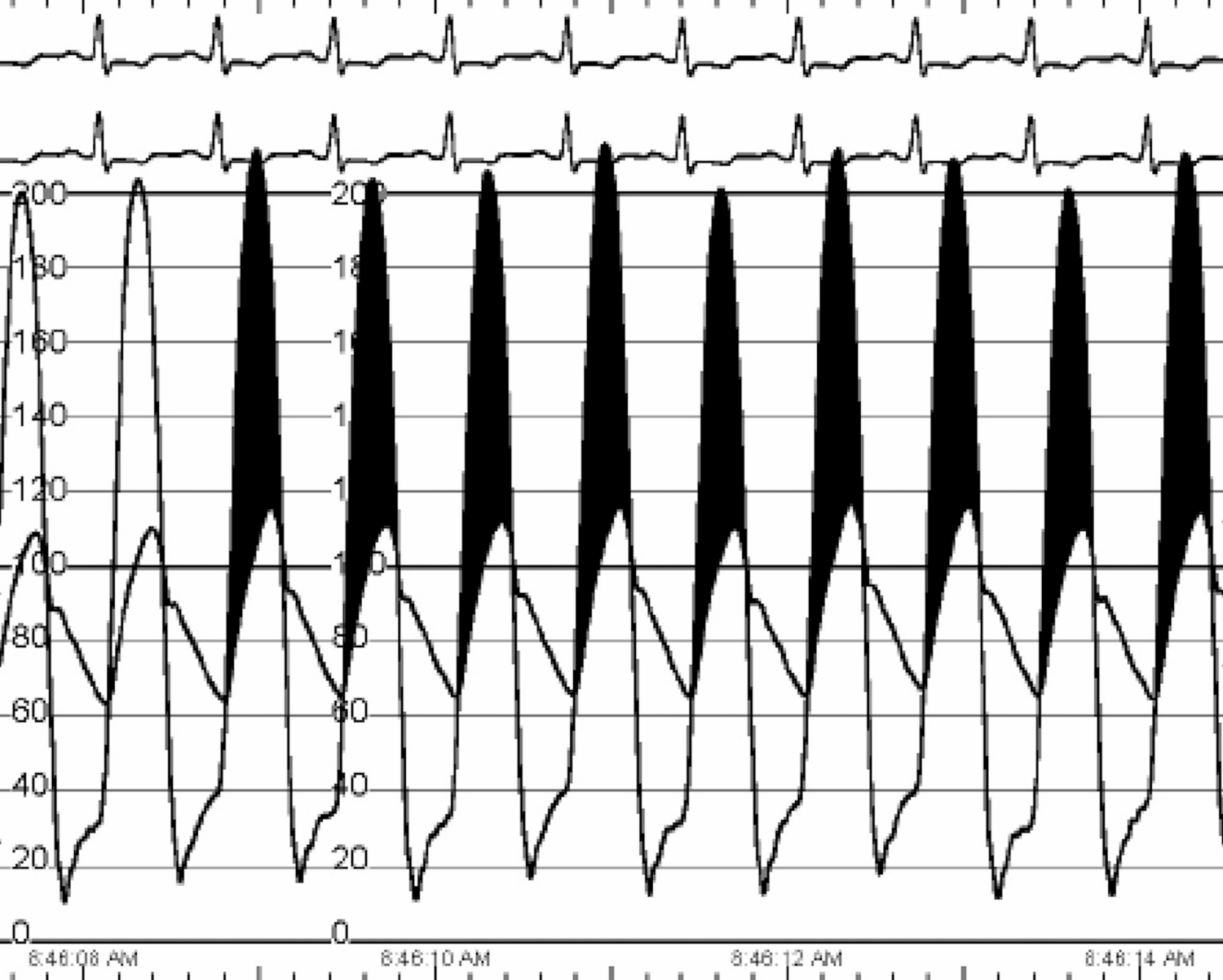
Left heart catheterization invasive hemodynamics exhibiting severe aortic stenosis (heart rate 91, mean gradient 66 mmHg, valve area 0.27 cm2, cardiac output 2.85).

**Figure 4:**
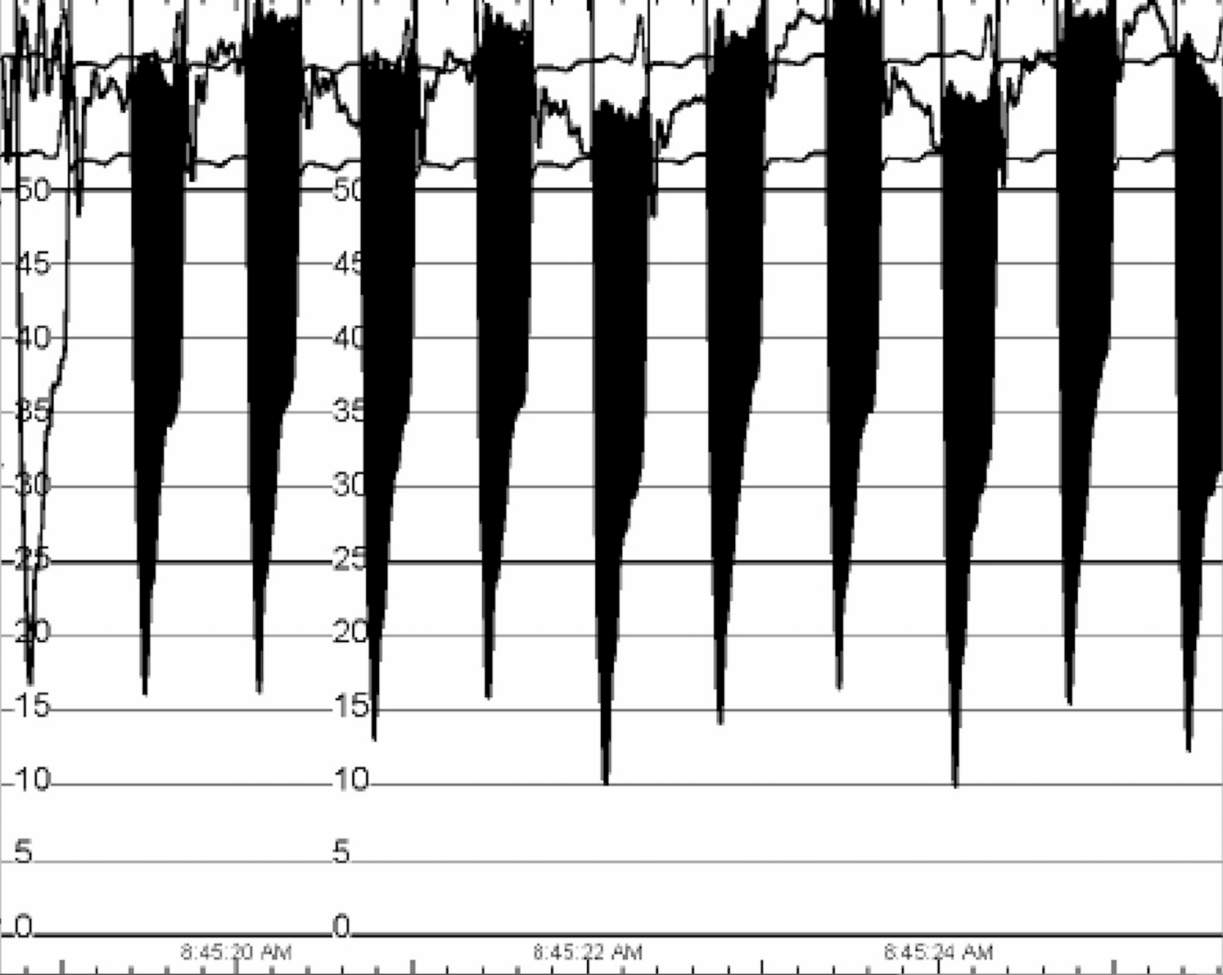
Left heart catheterization invasive hemodynamics exhibiting mitral stenosis (heart rate 91, mean gradient 28 mmHg, valve area 0.49 cm2, cardiac output 2.85).

LHC showed patent graft to the second diagonal artery (Figure 2), old complete stenosis of left main and left anterior descending artery with new circumflex artery occlusion. The LHC pressure tracings demonstrated aortic and MV stenosis (Figures 3, 4).

The patient developed cardiogenic shock during her hospital stay and was treated with inotropes followed by valve-in-valve transcatheter AVR (26 Evolut; Medtronic Inc., Minneapolis, MN). She was discharged home on guideline-directed medical therapy (GDMT) with carvedilol, losartan, furosemide, aspirin and Plavix.

## Discussion

TA is a diffuse inflammatory arteritis involving large vessels of the body. Coronary involvement in TA is not uncommon and can be divided into three types based on histopathology [4].Type I represents stenosis or occlusion of the coronary vessels. Type II involves arteritis of the coronary vessels and type III includes coronary aneurysms [5].

Occlusion of coronary vessels, representing 60%-80% of the cases, typically involves the ostial and proximal arteries and is the most common cause of mortality. In these patients, timely revascularization should be performed as prognosis for conservative management is poor [5,6]. Open surgical approach, including CABG, patch angioplasty or transaortic coronary ostial endarterectomy, is preferred as endovascular interventions have been associated with an increased risk of restenosis [7]. Although CABG is most commonly employed, currently there is no data to prove superiority of one procedure over the other.

In patients undergoing CABG, saphenous vein grafting (SVG) is recommended due to increased risk of inflammatory involvement of subclavian artery. However, the risk of graft occlusion, mostly involving the proximal anastomotic site, is still high with long-term graft patency limited to only 60% [8]. Thus, multiple studies have reported patients, similar to ours, undergoing repeat vascularization with percutaneous coronary intervention or CABG during their lifetime [9].

Valvular and aortic root involvement in TA is frequently seen, and aortic regurgitation (AR) is considered a significant risk factor for mortality in these patients. This is likely due to the development of chronic heart failure, as was the case in our patient. AVR and composite graft replacement (CGR) are the two surgical procedures available. Because CGR removes more inflamed tissue and decreases the risk of aortic dilation later on, it has shown to have less complications and a relatively long-term disease free status [10]. Since TA presents in the second to third decade of life, mechanical valves in patients undergoing AVR would be ideal. However, female patients in childbearing age may opt for bioprosthetic valves to avoid taking lifelong warfarin due to its risk of teratogenicity. Unfortunately, due to the relatively shorter life span of bioprosthetic valves as compared to mechanical valves, patients may present with recurrent aortic stenosis requiring repeat AVR. Moreover, in these patients, surgical management is challenging due to the need to manipulate friable and inflamed tissue with valve detachment and anastomotic aneurysms being the most common and life-threatening complications [11].

Our patient also had severe mitral regurgitation (MR) leading to MV annuloplasty. Studies have shown the incidence of MR in TA to be 3%, and it is usually reported in association with severe aortic disease [12]. The etiology however is not clear and may be related to inflammation from TA or from papillary muscle dysfunction from left ventricular dilatation secondary to AR. In severe cases of MR, however, surgical intervention is warranted to prevent complications like heart failure. Our patient underwent mitral annuloplasty and then developed mitral stenosis (MS). Despite the frequent occurrence of MS after MR repair, the phenomenon is not well studied. While surgical factors, e.g. use of an undersized annuloplasty ring or subvalvular tethering, can cause MS, organic causes from TA cannot be excluded.

Even though corticosteroids are considered the mainstay of treatment in TA, relapse rates with steroid monotherapy are high with remission rates only reaching 60% with the addition of non-biologic and biologic agents [13]. Our patient had been on a biologic agent for many years but still presented with significant cardiovascular involvement not amenable to medical intervention. This highlights the fact that more research needs to be done on how to manage patients presenting with a need for repeat surgical intervention.

## Conclusions

TA can lead to extensive and catastrophic cardiovascular complications. Even though our patient underwent multiple surgical interventions 10 years ago, she presented again with significant coronary and valvular disease. This highlights the relapsing nature of TA and the importance of controlling the underlying inflammation. This case discusses the various surgical options available and highlights the fact that significant uncertainty exists for the management of these patients when they present with relapsing coronary and vascular involvement. 
